# Differential Subplastidial Localization and Turnover of Enzymes Involved in Isoprenoid Biosynthesis in Chloroplasts

**DOI:** 10.1371/journal.pone.0150539

**Published:** 2016-02-26

**Authors:** Catalina Perello, Ernesto Llamas, Vincent Burlat, Miriam Ortiz-Alcaide, Michael A. Phillips, Pablo Pulido, Manuel Rodriguez-Concepcion

**Affiliations:** 1 Program of Plant Metabolism and Metabolic Engineering, Centre for Research in Agricultural Genomics (CRAG) CSIC-IRTA-UAB-UB, Campus UAB Bellaterra, 08193 Barcelona, Spain; 2 Université de Toulouse, CNRS, UMR 5546, BP 42617 Auzeville, 31326 Castanet-Tolosan, France; Instituto de Biología Molecular y Celular de Plantas, SPAIN

## Abstract

Plastidial isoprenoids are a diverse group of metabolites with roles in photosynthesis, growth regulation, and interaction with the environment. The methylerythritol 4-phosphate (MEP) pathway produces the metabolic precursors of all types of plastidial isoprenoids. Proteomics studies in *Arabidopsis thaliana* have shown that all the enzymes of the MEP pathway are localized in the plastid stroma. However, immunoblot analysis of chloroplast subfractions showed that the first two enzymes of the pathway, deoxyxylulose 5-phosphate synthase (DXS) and reductoisomerase (DXR), can also be found in non-stromal fractions. Both transient and stable expression of GFP-tagged DXS and DXR proteins confirmed the presence of the fusion proteins in distinct subplastidial compartments. In particular, DXR-GFP was found to accumulate in relatively large vesicles that could eventually be released from chloroplasts, presumably to be degraded by an autophagy-independent process. Together, we propose that protein-specific mechanisms control the localization and turnover of the first two enzymes of the MEP pathway in Arabidopsis chloroplasts.

## Introduction

Plant chloroplasts are active metabolic machines that fix carbon using the energy of sunlight to produce myriad compounds that support plant growth and development and contribute to their environmental adaptation. The enzymes that participate in these metabolic pathways are typically encoded by genes in the nuclear genome and hence they must be imported into plastids for biological activity. Complexes at the outer and inner chloroplast envelope membranes import most plastidial proteins into the stroma [1]. After proteolytic removal of the N-terminal plastid targeting sequence, the mature proteins with additional targeting signals are further directed to specific subplastidial compartments, including thylakoid membranes and lumen [2,3]. When normal activity ceases or environmental challenges such as excess light, temperature peaks, oxidative stress or nutrient starvation render the proteins inactive, protein quality control systems ensure their refolding (i.e. reactivation) or degradation to prevent the formation of toxic protein aggregates and maintain protein homeostasis in the chloroplast [4–6].

Isoprenoids are one of the most diverse groups of plant metabolites and comprise a variety of compounds with remarkable economic interest as medicinal drugs, pigments, aromas, chemicals, nutrients, and biofuels [7]. Thanks to the presence of plastids, plant cells have not one but two pathways to produce isopentenyl diphosphate (IPP) and dimethylallyl diphosphate (DMAPP), the universal metabolic precursors of isoprenoids [7,8]. Fungal and animal cells produce these precursors by the exclusive operation of the mevalonate pathway, also used by plant cells to synthesize the building blocks of cytosolic and mitochondrial isoprenoids. In the plastid, however, the biochemically unrelated methylerythritol 4-phosphate (MEP) pathway is used for the production of IPP and DMAPP for plastidial isoprenoids that function in photosynthesis (chlorophylls, carotenoids, tocopherols, prenylquinones), growth regulation (gibberellins, cytokinins, abscisic acid, strigolactones), and interaction with the environment (isoprene, monoterpenes, diterpenes). Although the advantages for plants of retaining two pathways are not fully understood [9], it is possible that the physical separation of the pathways facilitates the optimal supply of the metabolic precursors required in each cell compartment. The first steps of the MEP pathway involve the production of MEP from pyruvate and glyceraldehyde 3-phosphate via deoxyxylulose 5-phosphate (DXP). Following these steps, catalyzed by the enzymes DXP synthase (DXS) and DXP reductoisomerase (DXR), MEP is converted into a mixture of IPP and DMAPP in five additional enzymatic steps [10–12].

All seven MEP pathway enzymes are encoded by nuclear genes and imported into plastids [10,13]. Proteomic studies have identified all of them in the stromal fraction [14]. Computational analyses, however, led to propose that DXS and DXR might be additionally targeted to the thylakoid membrane or lumen [15,16]. Overexpression of any of these two enzymes in plants often results in an enhanced accumulation of MEP-derived isoprenoids [17–22], supporting the conclusion that they are important control points over flux in the MEP pathway [12,23–25]. Consistent with this regulatory role, the levels of both DXS and DXR enzymes are tightly regulated at multiples levels beyond the control of gene expression [23,26,27], including degradation by the stromal Clp protease [28–30]. Analysis of transgenic *Arabidopsis thaliana* lines producing a GFP-fused version of the DXS protein (*35S*:*DXS-GFP* lines) helped to understand the post-translational control of enzyme distribution, levels and activity within the plastid [31]. Fluorescence corresponding to the DXS-GFP fusion protein showed a spotted distribution in chloroplasts, likely due to the formation of protein aggregates [30,31]. The subplastidial localization and degradation pathways of the DXR protein, however, have not been explored yet. In this study we provide experimental evidence that Arabidopsis DXS and DXR proteins follow distinct pathways for protein turnover, which in the case of DXR appears to involve the formation of likely non-autophagic vesicles.

## Materials and Methods

### Plant material and growth conditions

All the *Arabidopsis thaliana* lines used in this work are in the Columbia background, including the *clpr1-2* mutant allele [28]. The line *pCAMBIA3300-PGL34-YFP* [32] was a kind gift of Felix Kessler (Université de Neuchâtel, Switzerland). Constructs *35S*:*DXS-GFP* [31], *35S*:*DXR-GFP* [31] and *35S*:*G11-GFP* [33] were used for *Agrobacterium*-mediated transformation of Arabidopsis plants. Homozygous lines containing a single T-DNA insertion were selected based on the segregation of the corresponding resistance marker. For experiments, seeds were surface-sterilized and sown on top of solid Murashige and Skoog (MS) medium (with no sucrose or vitamins) in Petri dishes. After stratification for at least 2 days at 4°C in the dark, plates were transferred to growth chambers at 22°C and illuminated with fluorescent white light (photon fluence rate of 80 μmol m^−2^ sec^−1^) for 16h/day (long-day photoperiod, LD). When indicated, the medium was supplemented with fosmidomycin (Sigma) or concanamycin A (Sigma). For treatments with fosmidomycin, plants were germinated and grown on media supplemented with the inhibitor (50 μM). Fosmidomycin resistance was assayed as described [34]. For treatment with concanamycin A, plants were germinated and grown for 4 days under LD on MS medium. Then, individual seedlings were transferred to MS plates supplemented with 10 μM concanamycin A and incubated in the dark for 24h. Control seedlings were transferred to non-supplemented MS medium. For incubation in the dark, the plates were covered with several layers of aluminum foil. *Nicotiana benthamiana* plants used for transient expression experiments were grown on soil in the greenhouse at 18–28°C under LD conditions.

### Transient expression assays

Cultures of *Agrobacterium tumefaciens* EHA 105 cells transformed with constructs to produce DXS-GFP, DXR-GFP, or G11-GFP [31,33] were used for agroinfiltration of leaves from 4 to 6 week-old *N*. *benthamiana* plants using the syringe method [35]. To prevent silencing, leaves were co-infiltrated with an *Agrobacterium* strain transformed with a vector expressing the HC-Pro silencing suppressor [35]. A 1:1 mixture of the two cultures was agroinjected in the abaxial part of several leaves. Then, plants were left in the greenhouse and leaf samples were collected at different timepoints after injection (from day 1 to day 7) for further analyses.

### Analysis of transcript levels

RNA for quantitative PCR analysis was obtained from 30-day-old plants. The full rosette was harvested by grinding in liquid nitrogen, and RNA was obtained using the Qiagen RNeasy Plant kit from approximately 100 mg tissue (fresh weight). One μg total RNA was used in 20 μL reverse transcriptase reactions (Roche) using manufacturer’s instructions and a poly dT_18_V anchored primer at 48°C. cDNA synthesis reactions were halted after one hour by heating at 70°C and then diluted 1:10 (v/v) with pure water. One μL was used as template in a PCR that also included 10 μL 2X SYBR Green mix (Roche), 0.6 μL each forward and reverse primers (10 μM), and pure water for a final volume of 20 μL. Primers DXR-F (5’- A G T A G C G G A T G C G T T G A A G C) and DXR-R (5’- G C G G A T G A A T G A C A A T C T C T A T A T C G) were used in these experiments. cDNA loading in individual reactions was normalized to the levels of *APT1* and *RP2ls* genes, whose sequence and stability under these conditions were previously reported [36]. Six individual plants were used for each transgenic or wild type line and each biological replicate was analyzed in three technical replicates for each gene of interest or normalizer. Relative fold calculations were performed using the efficiency corrected model [37].

### Chloroplast isolation and membrane fractionation

Chloroplasts were isolated from 10 day-old-seedlings as described [28] and further fractionation was performed as indicated [38,39]. Briefly, chloroplasts were hypertonically lysed in 0.6 M sucrose supplemented with 0.5% (w/v) protease inhibitor cocktail (Sigma). Stromal fraction was collected after centrifugation at 100,000 x*g*. The membrane pellet was resuspended in the same buffer and centrifuged again to prevent stromal contamination. A Potter-Elvehjem homogenizer was used to resuspend the chloroplast membranes in 1 mL TED buffer (50 mM Tricine pH 7.5, 2 mM EDTA, 2 mM dithiothreitol). Subsequent separation was performed in sucrose density gradients as described [38,39] but using a half of the volumes. Native protein extracts from whole seedlings were used for the separation of soluble and insoluble (with protein aggregates) fractions as described [30].

### Immunoblot analysis of protein levels

Protein extracts from chloroplast fractions or whole plants were obtained as described above and elsewhere [31] and directly used for immunoblot analysis or proteinase K accessibility assays as described [30]. Antibodies raised against DXS and DXR [31], GFP (Invitrogen), plastome-encoded ClpP1 [28], and chloroplast-imported proteins AtpB, ClpB3, PsbA, and Tic40 (Agrisera) were used diluted 1:500 for DXS and Tic40, 1:1,000 for ClpP1, 1:2,000 for GFP, AtpB and PsbA, 1:3,000 for ClpB3, and 1:6,000 for DXR. Chemiluminescent signals were visualized using a LAS-4000 image analyzer (Fujifilm) and quantified with Quantity One (Bio-Rad).

### Confocal imaging

Subcellular localization of GFP fusion proteins was observed by direct examination of plant tissue with a Leica TCS 4D Confocal Laser Scanning Microscope. GFP fluorescence was detected using a BP515-525 filter after excitation with blue light at 488 nm. Chlorophyll autofluorescence was detected using a LP590 filter after excitation with green light at 568 nm. Stacks of acquired images were processed with Imaris 6.1.5 (Bitplane) to achieve three dimensional images.

### Transmission electron microscopy (TEM) immunogold labelling

Transgenic *35S*:*DXR-GFP* seedlings were germinated and grown on MS plates for 3 days under LD conditions. Then, cotyledon samples were collected and immediately fixed as described [40]. Briefly, freshly cut cotyledons were dissected in fresh fixative solution (1.25% (v/v) glutaraldehyde and 2% (v/v) formaldehyde in sodium 0.05 M phosphate buffer pH 7.5), and placed under gentle vacuum for 5 cycles of 2 min each. Then, samples were kept in fixative solution for 2h at room temperature. After 3 washes of 10 min with 0.05 M sodium phosphate buffer pH 7.5, dehydration was carried out in a series of 10 min washes with 30%, 50%, 70%, 80%, 95% and 100% ethanol. For infiltration, a mixture of LR White acrylic resin (Sigma) and 100% ethanol at proportions 1:2, 1:1, 3:1, and 1:0 (1h each) was used. Following several resin changes for 3 days at 4°C, sample embedding was performed by polymerization for 24 h at 50°C in 0.3 ml gelatin capsules.

Ultrathin (100 nm) sectioning and immunogold labelling was carried out as described [41] using anti-DXR or pre-immune sera. Silver enhancement was carried out for 3–5 min at 20°C with intenSE (Amersham-Pharmacia Biotech) following manufacturer’s instructions. Post-staining employed 2% uranyl acetate aqueous solution for 2 min followed by 2 quick rinses in distilled water. Observations were made at 80 kV using a Jeol 1200 EX transmission electron microscope equipped with a Gatan Bioscan camera to capture digital pictures.

## Results

### Arabidopsis DXS and DXR are stromal proteins that can also be found in insoluble and membrane fractions

**Fig 1:**
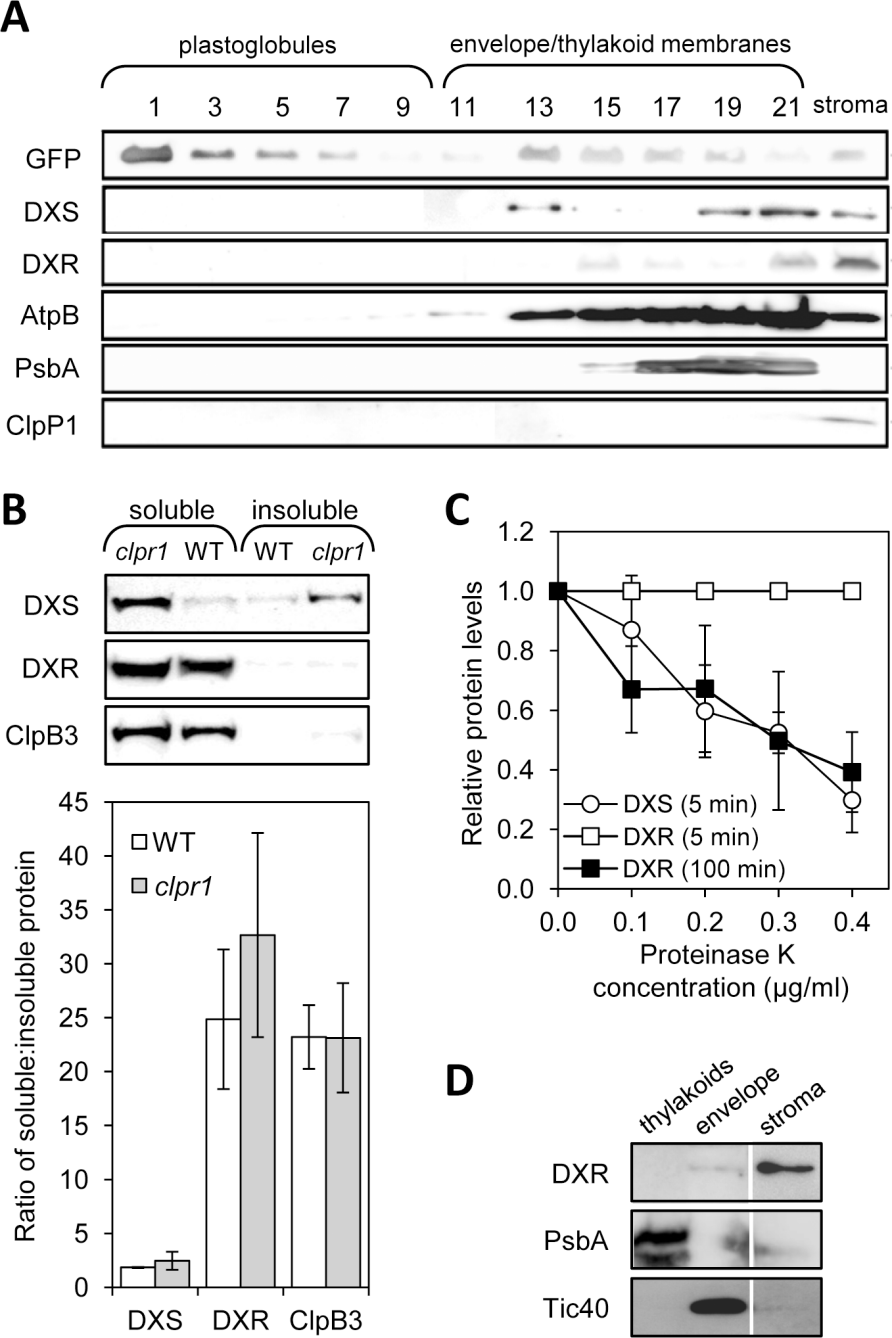
Immunoblot analysis of chloroplast subfractions and protein stability. (**A**) Chloroplasts isolated from transgenic plants overexpressing PGL34-YFP were used to separate soluble (stromal) and membrane fractions. The latter were loaded in a sucrose density gradient and separated by ultracentrifugation. Proteins contained in 35 μl of sequential fractions collected from the top of the gradient or from the stromal sample were separated by SDS-PAGE and transferred to a membrane for immunoblot analysis with antibodies against GFP (to detect PGL34-YFP) or the indicated endogenous proteins. (**B**) Immunoblot analysis of the distribution of DXS and DXR proteins in soluble and insoluble fractions isolated from native protein extracts of wild type and *clpr1* mutant seedlings. The graph represents mean ± SEM of the ratios of soluble vs. insoluble protein levels in n = 3 independent experiments. (**C**) Quantification of DXS and DXR protein levels after immunoblot analysis of wild type protein extracts incubated with the indicated concentrations of proteinase K for the indicated times. Mean ± SEM of n = 4 independent experiments are shown. (**D**) Immunoblot analysis of the distribution of DXS in envelope and thylakoid membranes isolated from wild type chloroplasts. A lane corresponding to the stromal fraction is also shown. The same extracts were incubated with antibodies against marker proteins of the envelope (Tic40) and the thylakoid membranes (PsbA). Representative blots are shown in all cases.

The first two enzymes of the MEP pathway, DXS and DXR, have been proposed to contain bipartite sorting sequences for subplastidial targeting to the thylakoid membrane or lumen [15,16]. However, no experimental evidence is currently available to support this claim, as proteomic studies identified both enzymes in the stroma [14]. As a first step to test the computational analysis predictions, we investigated whether the endogenous Arabidopsis enzymes were associated to thylakoids or associated structures such as plastoglobules by separating them using flotation centrifugation with sucrose density gradients [32] and then analyzing the presence of DXS and DXR in the fractions by immunoblot analysis (Fig 1A). Chloroplasts were isolated from transgenic plants producing the plastoglobule marker PGL34-YFP [32] and used for membrane fractionation, protein extraction, and immunoblot analysis with antibodies against DXS and DXR as described [30,31,38]. The same fractions were also analyzed for the presence of control proteins known to be localized in plastoglobules (PGL34-YFP), embedded in the thylakoid membrane (PsbA), associated to the stromal side of the thylakoid membrane (AtpB), or found in the stroma (ClpP1). Both DXS and DXR were found in the soluble (stromal) fraction, as expected. However, DXS and, to a lower extent, DXR proteins were also detected in fractions corresponding to membrane-containing structures other than plastoglobules (Fig 1A).

A recent work showed that DXS is prone to aggregation [30]. DXS aggregates become insoluble and associate to chloroplast membrane fractions until they are either solubilized by specific disaggregating chaperones or eventually degraded by the stromal Clp protease complex [30]. In wild type Arabidopsis plants growing under normal conditions, the proportion of soluble to insoluble (i.e. membrane-associated) DXS protein is close to 2:1 (Fig 1B). When Clp-mediated degradation of DXS is compromised, total DXS protein levels increase but the proportion of soluble to insoluble protein remains virtually unchanged because disaggregating chaperones such as ClpB3 also accumulate to relief protein folding stress (Fig 1B) [28–30,42,43]. Thus, Clp-defective mutants such as *clpr1* show increased levels of both ClpB3 and DXS proteins but no changes in the soluble to insoluble ratio (Fig 1B). DXR levels are also upregulated in *clpr1* plants, suggesting that this protein might also be a Clp protease client [28]. But unlike DXS, DXR is not prone to aggregation [30] and it remains mostly soluble in wild type plants and *clpr1* mutants, showing a proportion of around 25:1 of soluble to insoluble protein (Fig 1B). Most interestingly, the rate of degradation of DXR by the Clp protease appears to be similar to that for DXS [28] whereas a 20-fold longer incubation with proteinase K was required to degrade DXR at the same rate than DXS in wild type plant extracts (Fig 1C). Together, the results suggest that the association of DXS to chloroplast membrane fractions might be an unspecific effect resulting from the formation of insoluble protein aggregates (particularly after stress episodes). In the case of DXR, most protein is found soluble in stromal fractions (i.e. non-aggregated) but poorly accessible to unspecific proteolytic degradation (as deduced from proteinase K protection assays).

### Analysis of GFP-fusion proteins confirms a differential distribution of DXS and DXR within chloroplasts

**Fig 2:**
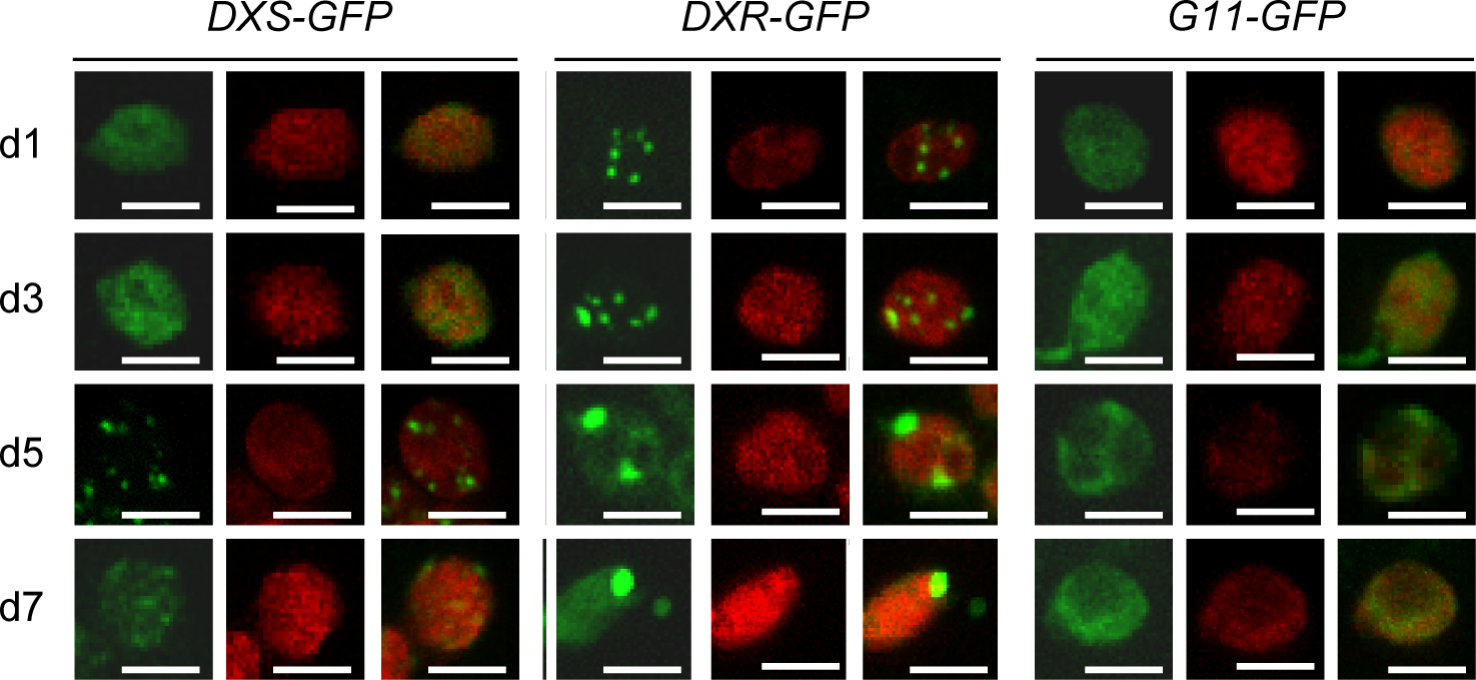
Distribution of GFP-tagged isoprenoid enzymes in chloroplasts of agroinfiltrated *N*. *benthamiana* leaves. Images show representative mesophyll chloroplasts from leaves collected at different days (from 1 to 7) after agroinfiltration with the indicated constructs. For each construct, GFP fluorescence (left columns), chlorophyll autofluorescence (middle columns) and merged images (right columns) are shown. Bars, 5 μm.

To further investigate the different subplastidial distribution of DXS and DXR, we analyzed the localization of full-length versions of the proteins fused to GFP at different time points after agroinfiltration of *Nicotiana benthamiana* leaves with the corresponding *35S*:*DXS-GFP* and *35S*:*DXR-GFP* constructs [31]. As a stromal protein control we used the isoprenoid enzyme geranylgeranyl diphosphate synthase 11 (GGPPS11 or G11), previously found to be exclusively located in the stroma by both proteomic approaches and confocal microscopy analysis of G11-GFP fluorescence [14,44]. As expected, chloroplasts of *N*. *benthamiana* leaves agroinfiltrated with the *35S*:*G11-GFP* construct [33] showed a fairly homogeneous distribution of fluorescence within the chloroplast at all time points analyzed, from day 1 (d1) to day 7 (d7) after agroinfiltration (Fig 2). G11-GFP fluorescence was also detected in stromules (Fig 2), consistent with its reported localization in the stroma.

Stromal fluorescence was also detected for DXS-GFP and DXR-GFP at all timepoints analyzed. However, these two fusion proteins were also found to form fluorescent corpuscles (Fig 2). In the case of DXS-GFP, fluorescence was predominantly localized in the stroma at early timepoints after agroinfiltration. Later on (d5), it showed a spotted distribution like that reported for the DXS-GFP fusion protein in stably transformed Arabidopsis plants (Fig 2) [30,31]. By d7, fluorescent spots were fainter and a more general distribution of the DXS-GFP protein in chloroplasts was observed (Fig 2). This is consistent with our current understanding that DXS-GFP protein overaccumulation could lead to the formation of protein aggregates (fluorescence spots). Subsequent up-regulation of disaggregating chaperones such as ClpB3 would eventually contribute to remove the DXS-GFP clumps, resulting in a more disperse fluorescence [30]. A distribution in intraplastidial speckles was also observed for DXR-GFP, but in this case small fluorescent spots were already detected during the first stages (d1-d3) following agroinfiltration (Fig 2). Then, DXR-GFP fluorescence concentrated in a few large bodies in the chloroplast (Fig 2). Some fluorescent bodies lacking chlorophyll were also detected outside the chloroplast, particularly at late timepoints (d7). The following experiments were designed to study the nature of these bodies.

### DXR-GFP accumulates in large fluorescent bodies that can eventually leave the chloroplast

**Fig 3:**
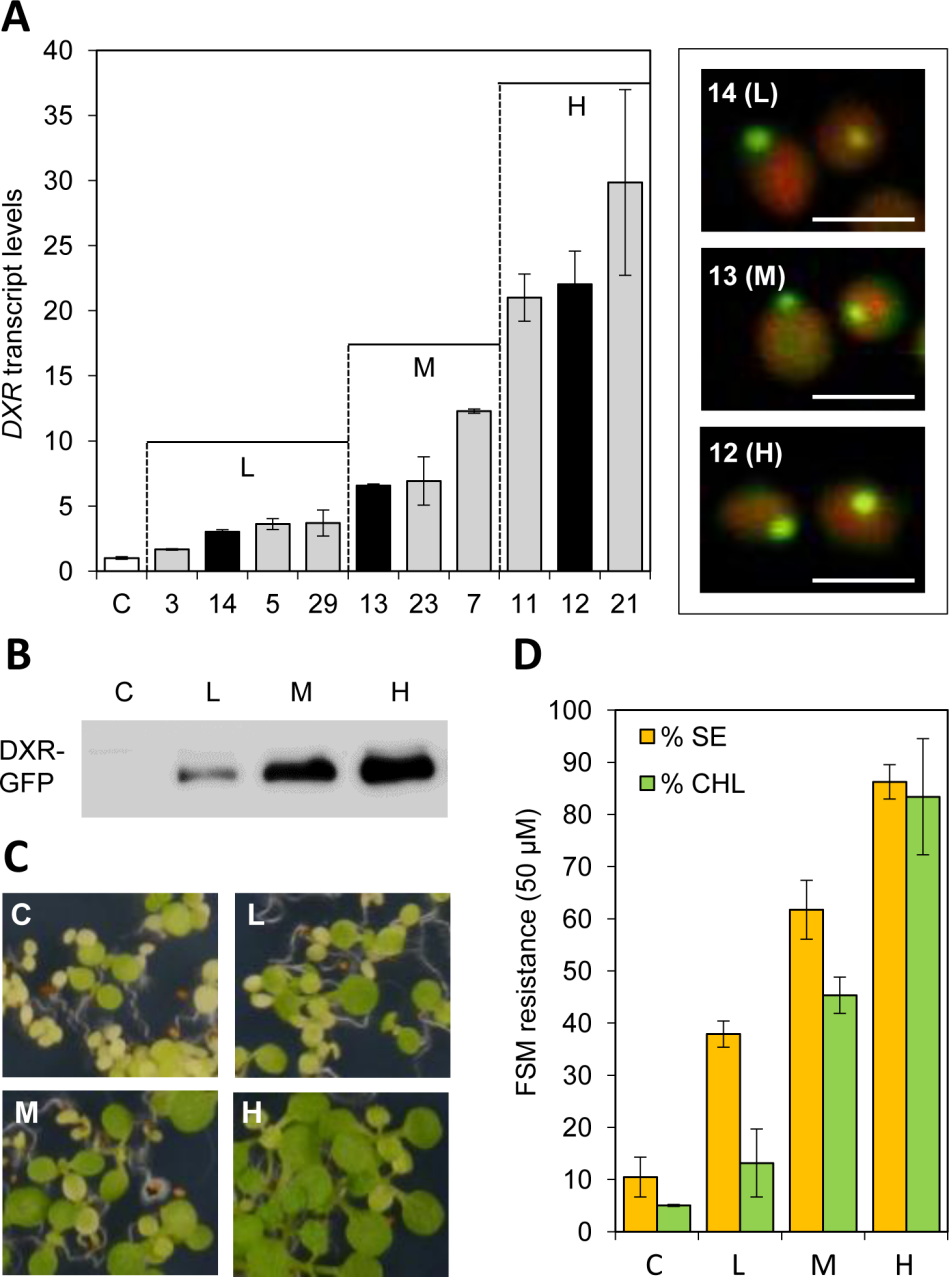
Characterization of transgenic Arabidopsis lines producing DXR-GFP. (**A**) Relative levels of *DXR* transcripts in 30-day-old soil-grown wild type plants (C, white column) and *35S*:*DXR-GFP* lines (grey and black columns) (n = 6 per group). The box on the right shows images of merged chlorophyll and GFP fluorescence signals in chloroplasts from the guard cells of lines representative of low (L), medium (M) and high (H) transgene expression levels (black columns). (**B**) Immunoblot analysis of DXR-GFP levels with an anti-GFP antibody in protein extracts (10 μg) from 10-day-old seedlings of the indicated lines. (**C**) Representative pictures of seedlings of the indicated lines germinated and grown for 10 days in the presence of fosmidomycin (50 μM). (**D**) Quantification of the phenotype observed in (C) as the percentage of seedling establishment (SE) and chlorophyll content (CHL) in the presence of fosmidomycin relative to those in the absence of inhibitor.

To further investigate the nature of DXR-GFP bodies, the *35S*:*DXR-GFP* construct was used to stably transform Arabidopsis plants of the Columbia accession (Fig 3). Transgenic lines with different levels of transgene expression were obtained, ranging from 2-fold to 30-fold higher levels of DXR-encoding transcripts compared to untransformed controls (Fig 3A). Representative lines with low (L), medium (M) or high (H) levels of transgene expression were next selected for immunoblot analysis of DXR-GFP protein levels using a GFP-specific antibody. As shown in Fig 3B, a good correlation between transcript and protein levels was found. To evaluate whether the recombinant protein accumulated in transgenic lines was enzymatically active, we used an indirect assay to estimate DXR activity *in vivo* based on quantifying plant resistance to fosmidomycin (FSM), a competitive inhibitor of DXR activity [34,45,46]. Blockage of DXR activity with FSM causes a concentration-dependent inhibition of seedling establishment (SE, defined as the production of true leaves that can support further plant development) and chlorophyll accumulation [19,47]. Both parameters can be used as a quantitative measure of FSM resistance [34]. After germination and growth in the presence of 50 μM FSM, only a low proportion of untransformed control plants were able to produce some chlorophyll and develop rudimentary true leaves (Fig 3C). This proportion significantly (p<0.05) increased in transgenic lines (Fig 3C), showing a strong correlation between DXR-GFP levels (Fig 3B) and FSM resistance estimated as SE rates and chlorophyll content in the presence of inhibitor (Fig 3D). We therefore concluded that at least a fraction of the DXR-GFP protein produced in the transgenic lines is enzymatically active. This is consistent with our previous conclusion that DXR mostly accumulates in a non-aggregated form (Fig 1).

**Fig 4:**
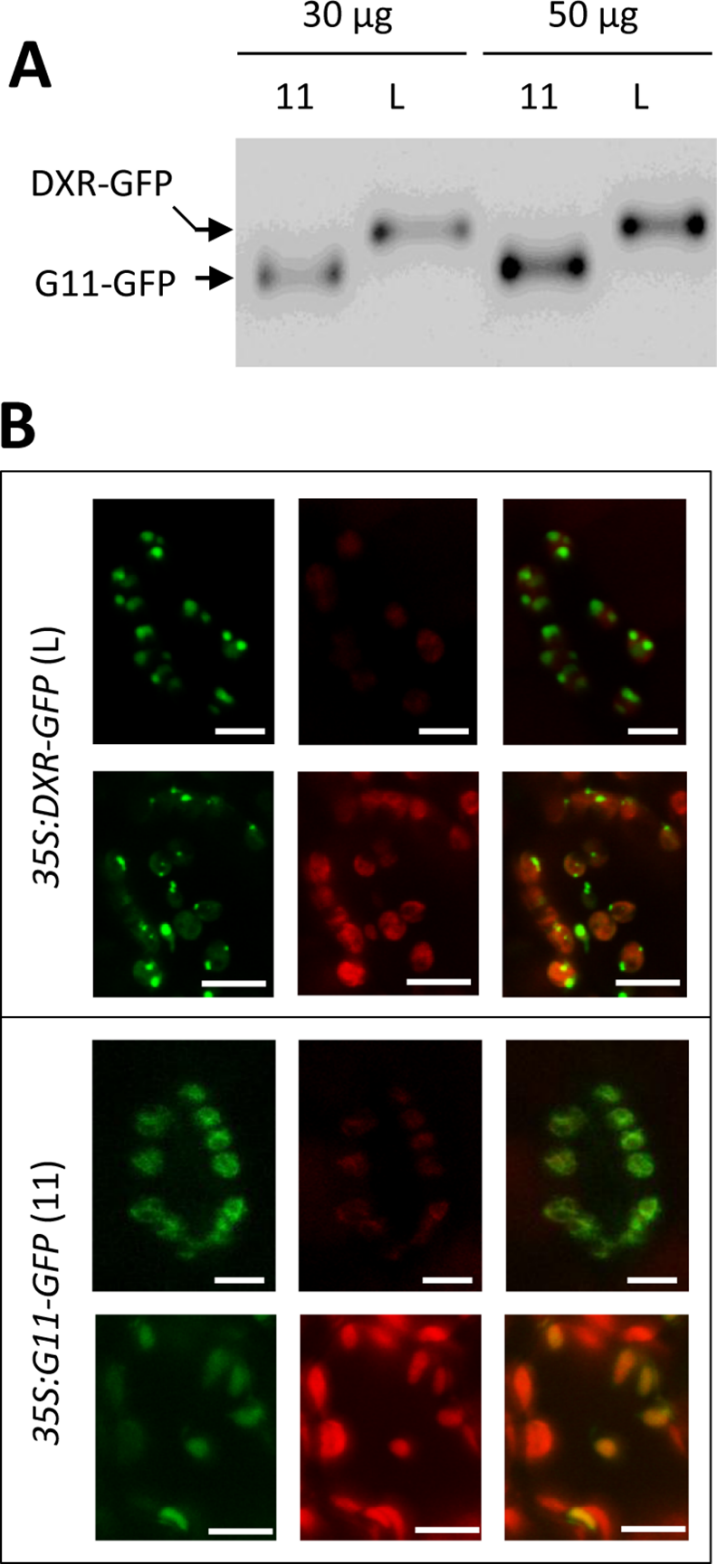
Differential distribution of DXR-GFP and GGPPS11-GFP proteins in chloroplasts of transgenic Arabidopsis plants. (**A**) Immunoblot analysis of protein extracts from *35S*:*DXR-GFP* (L line) and *35S*:*G11-GFP* (11) plants with an anti-GFP antibody. Results with two different protein extract amounts are shown to illustrate that these lines have very similar levels of the corresponding GFP-tagged protein. (**B**) Representative images of stomata (upper rows) and mesophyll cells (lower rows) from leaves of the plants analyzed in (A). Images show GFP fluorescence (left columns), chlorophyll autofluorescence (central columns), or both (right columns). Bars, 5 μm (stomata) and 10 μm (mesophyll).

Despite the clear differences between the levels of recombinant DXR-GFP protein accumulating in the selected Arabidopsis L, M, and H lines, all of them showed a punctate pattern of GFP fluorescence (Fig 3A) similar to that observed in *N*. *benthamiana* leaves transiently expressing the same reporter (Fig 2). Chlorophyll-lacking fluorescent spots were also observed in the periphery of chloroplasts in all lines (Fig 3A). To test whether this distribution could be an artifact caused by the unspecific overaccumulation of any plastid-targeted GFP fusion protein, Arabidopsis plants were transformed with the *35S*:*G11-GFP* construct and the levels of G11-GFP protein were analyzed in the resulting transgenic lines [33]. The maximum accumulation of G11-GFP obtained by this strategy was similar to that of DXR-GFP in L lines, as estimated by immunoblot analysis with a GFP-specific antibody (Fig 4A). Comparison of lines showing similar levels of these two recombinant proteins confirmed that G11-GFP fluorescence was uniformly distributed in the stroma of stomata and mesophyll chloroplasts whereas DXR-GFP fluorescence accumulated in large speckles both inside and outside chloroplasts (Fig 4B). While overproduction of a GFP-tagged protein can certainly alter the stoichiometry of the protein complement in the chloroplast and eventually cause artifacts, the observation that only the accumulation of DXR-GFP proteins (but not of very similar levels of G11-GFP) triggers the formation of large corpuscles indicates that this is a specific effect of the DXR protein.

**Fig 5:**
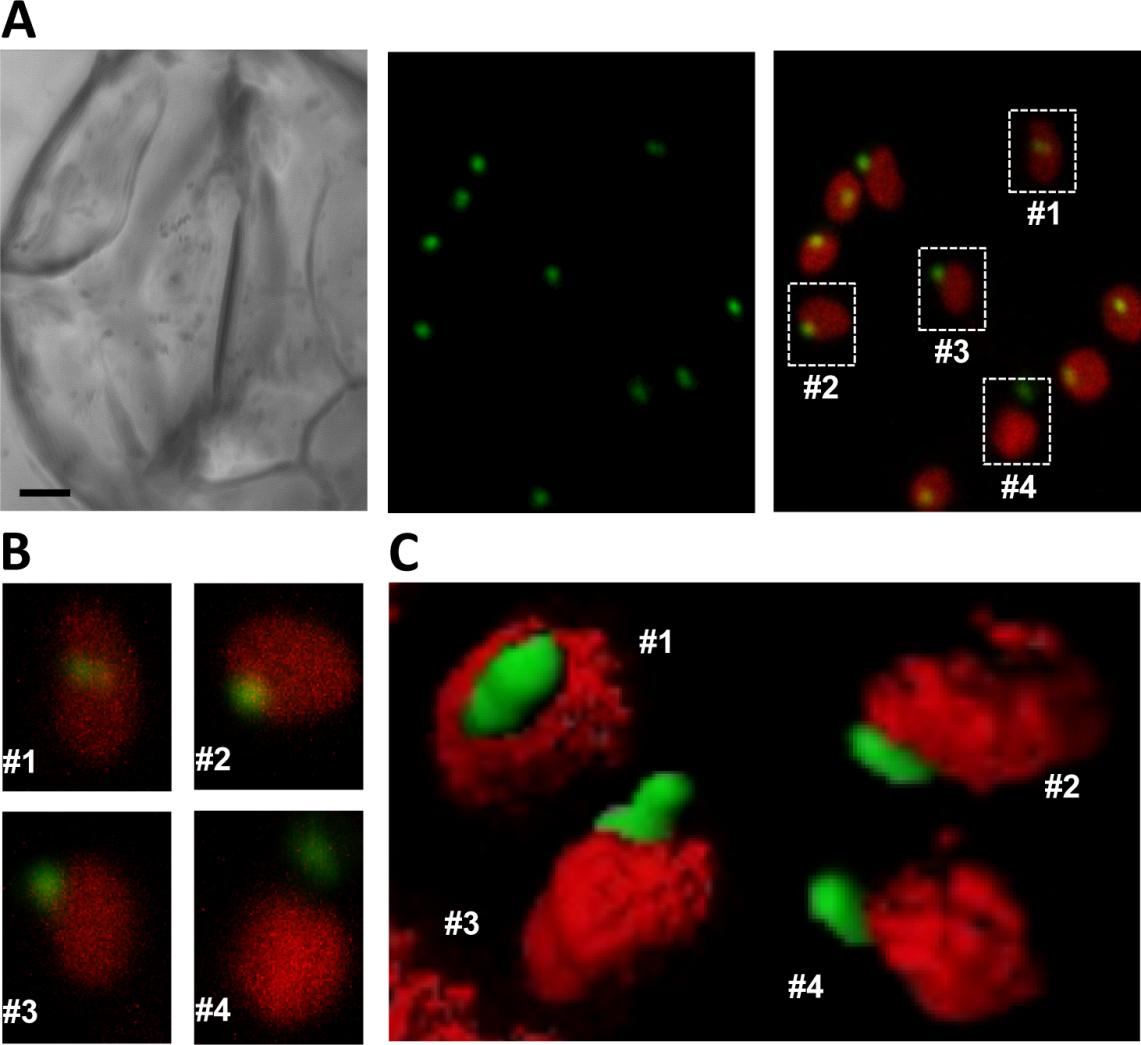
Differential localization of DXR-GFP bodies inside and outside chloroplasts. (**A**) Guard cells of a *35S*:*DXR-GFP* (H line) plant. The pictures show a bright field image (left panel), GFP fluorescence (central panel), and merged GFP and chlorophyll fluorescence (right panel). Bar, 2 μm. (**B**) Magnification of the chloroplasts boxed in (A). (**C**) Reconstructed 3D images of representative chloroplasts at the indicated phases of DXR-GFP vesicle development.

**Fig 6:**
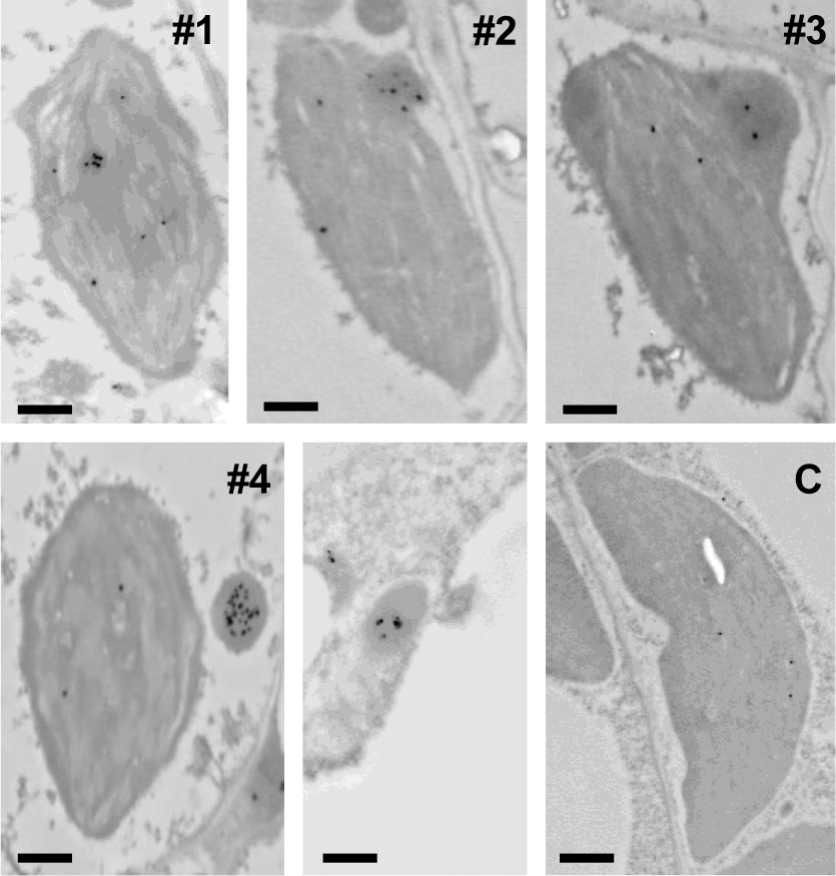
Immunolocalization of DXR-GFP in vesicles. Cross-sections of cotyledons from transgenic plants expressing DXR-GFP (H line) and untransformed wild type controls (C) were used for immunogold labelling with anti-DXR serum and observed at the transmission electron microscopy (TEM) level. Numbers indicate phases of DXR-GFP vesicle development in the transgenic lines (the unnumbered panel shows an isolated vesicle). ar, 0.5 μm.

Fluorescent bodies containing DXR-GFP were found inside the chloroplast (what we refer to as phase #1) in the periphery of the organelle (#2), protruding from the chloroplast (#3), or completely separated from it (#4) (Fig 5). All four different phases could be observed even within the same cell (Fig 5A). Serial confocal sectioning showed that DXR-GFP bodies were elongated and confirmed that they were virtually devoid of chlorophyll autofluorescence (Fig 5B and 5C), suggesting that they might be formed by stromal content. Immunolabeling of DXR-GFP in *35S*:*DXR-GFP* (H) plants using polyclonal anti-DXR antibodies followed by transmission electron microscopy (TEM) identified the same phases in mesophyll cells (Fig 6). While no labeling was found in transgenic samples incubated with preimmune serum, TEM observations of *35S*:*DXR-GFP* (H) sections incubated with anti-DXR detected labeling of actual vesicles containing the recombinant protein. Consistent with the confocal microscopy data (Fig 5), these vesicles had an estimated size of around 0.5–1 μm in diameter and displayed an electron density similar to that of the stroma (Fig 6). Also in agreement with confocal microscopy data, most of the vesicles in TEM sections were observed inside chloroplasts (i.e. in phase #1), some were found in phases #2 and #3, and only a few were located free in the cytoplasm (#4). We therefore concluded that these phases could correspond to sequential stages in a pathway to expel DXR-GFP from chloroplasts, i.e. they would be formed by engulfing stromal DXR-GFP enzymes and then they would be transported to the plastid envelope and released from chloroplasts.

DXR immunolocalization experiments in untransformed (wild type) controls detected only occasional labeling and no distinctive vesicles in chloroplasts (Fig 6). However, separation of envelope and thylakoid membranes isolated from wild type chloroplasts followed by immunoblot analysis of DXR and marker proteins (Tic40 for the envelope and PsbA for thylakoids) showed that a fraction of the endogenous DXR enzyme was associated with envelope membranes (Fig 1D). These results, together with the observed resistance of DXR in protease protection assays despite its solubility (Fig 1C), suggest that some of the endogenous DXR enzyme might indeed be found inside vesicles formed by stromal material engulfed by envelope membranes.

### Excess DXR might be removed from chloroplasts by a process likely independent of autophagy

**Fig 7:**
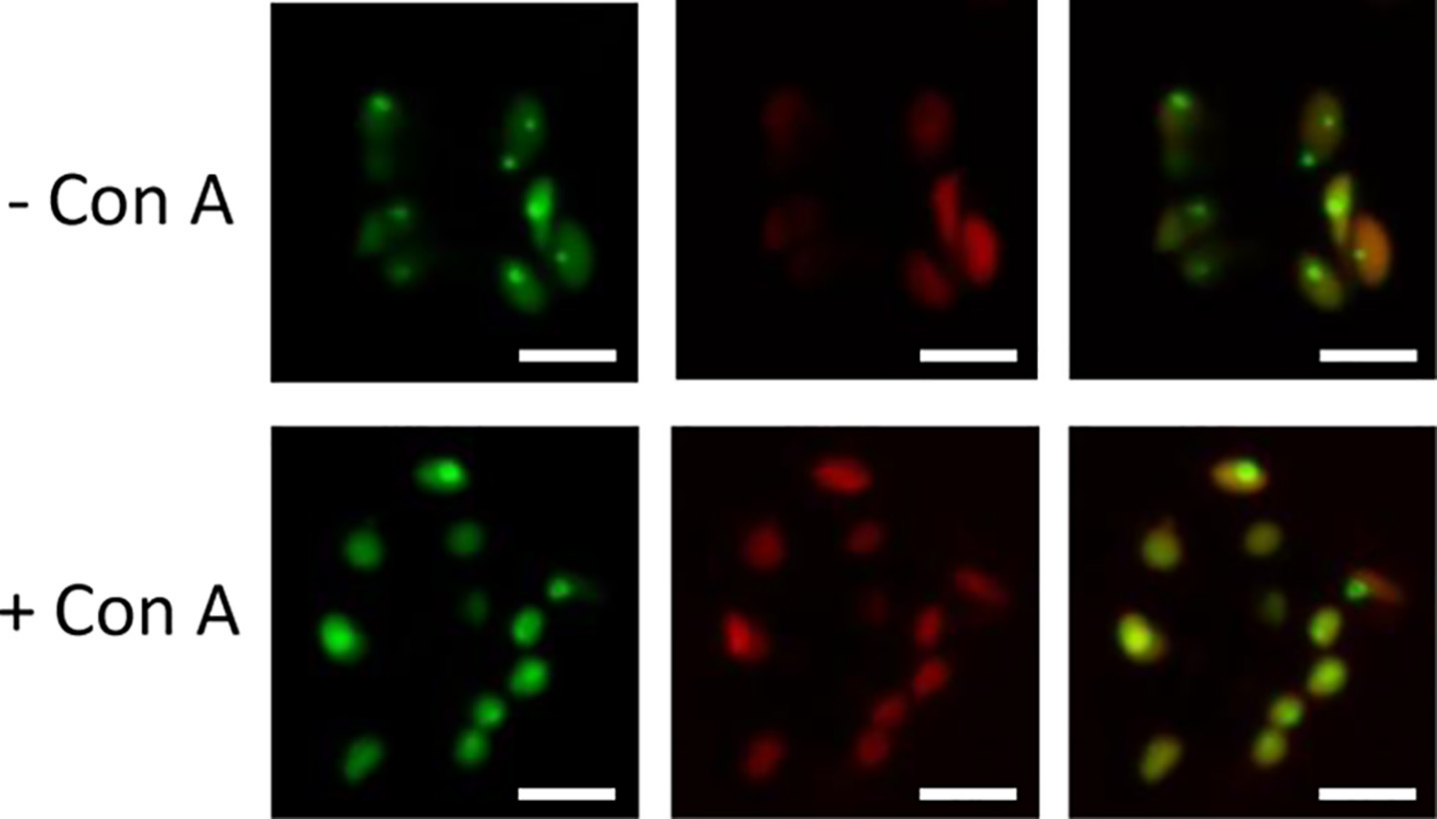
Effect of concanamycin A on DXR-GFP localization. Pictures show representative images of GFP fluorescence (left columns), chlorophyll autofluorescence (central columns), or both (right columns) in guard cells of *35S*:*DXR-GFP* (H line) plants either exposed (+) or not (-). to 10 μM concanamycin A for 24 h. Bars = 5 μm.

The features of DXR-GFP vesicles were reminiscent of previously identified Rubisco-containing bodies (RCBs) and starch granule-like structures (SGLSs), which are released from the chloroplast and delivered to the vacuole for degradation by autophagy [48–50]. To investigate whether the vesicles containing DXR-GFP could be of autophagic nature we used concanamycin A, an inhibitor that promotes an accumulation of autophagic bodies inside the vacuole [48,51]. Transgenic *35S*:*DXR-GFP* (H) lines were germinated and grown for 4 days under LD conditions (16h light and 8h dark) on solid MS medium and then individual seedlings were transferred to fresh medium either supplemented or not with 10 μM concanamycin A. Plates were incubated for 24h in the dark before analyzing the presence of DXR-GFP fluorescence by confocal fluorescence microscopy. As shown in Fig 7, concanamycin A-treated plants did not show more fluorescent vesicles in vacuoles but instead a more diffused distribution of DXR-GFP inside chloroplasts. A similar effect had been previously reported for CV (for Chloroplast Vesiculation) containing vesicles (CCVs), which are normally expelled from chloroplasts by an autophagy-independent process [52]. We therefore conclude that a pathway independent of autophagy might be responsible for the eventual degradation of DXR-GFP bodies once released from the chloroplast.

## Discussion

While all MEP pathway enzymes have been identified in the stroma by proteomic studies [14], *in silico* predictions [15,16] and the experimental data reported here show that DXS and DXR can also associate with membrane structures in the chloroplast. Furthermore, a differential distribution of DXS and DXR in non-stromal, particulate fractions was observed by immunoblot analysis of the endogenous enzymes in chloroplast membrane (i.e. insoluble) subfractions (Fig 1) and by fluorescence detection of GFP-tagged versions either transiently (Fig 2) or stably (Fig 3) [31] expressed. The localization of the same protein in different chloroplast subcompartments appears to be quite common [14,53]. This might be achieved by the use of different sorting signals in the protein sequence or by interaction with other protein partners that are delivered to different plastidial locations. While stroma-localized DXS and DXR could fulfill the expected role of these enzymes in the first two consecutive steps of the MEP pathway, a proportion of both proteins might be delivered to other subplastidial locations using distinct mechanisms. Based on the results shown here, we conclude that the prediction that DXS and DXR might be targeted to thylakoids upon import into chloroplasts [15,16] is unlikely. Instead, the different propensity of these two MEP pathway enzymes to aggregate and become insoluble might explain their distinct pattern of subplastidial distribution and membrane association.

Most DXS is found soluble in the stroma, but this enzyme is prone to become misfolded (i.e. inactive) and to aggregate even under normal growth conditions (Fig 1) [30]. It is therefore possible that the DXS proteins detected here in insoluble chloroplast fractions correspond to aggregates of inactive protein that unspecifically bind to chloroplast membranes. These aggregates, observed as fluorescent speckles in cells expressing the DXS-GFP reporter protein (Fig 2) [31], can be eventually solubilized and the protein degraded by the Clp protease complex [30]. While DXR might also be a target for the Clp protease [28], the mechanism for excess protein removal appears to be different. Unlike DXS, DXR appears to be a very stable protein. Is more resistant than DXS to degradation by unspecific proteases (Fig 1C) or by endogenous (likely Clp) proteases after a heat shock [31], and it remains in the soluble (stromal) fraction even under stress conditions that promote general protein aggregation (Fig 1) [30]. We observed here that an extra production of recombinant DXR-GFP protein appears to result in their storage in large elongated bodies or vesicles. Even though we have only observed these vesicles in transgenic lines expressing a GFP-tagged DXR enzyme under a strong constitutive promoter (*35S*), the fact that they are not formed in plants overexpressing the G11-GFP protein at similar levels (or in many other transgenic lines producing GFP fusion proteins in chloroplasts; http://podb.nibb.ac.jp/Organellome/) suggests that they are not an artifact. The observation that the vesicles are formed even when levels of DXR-GFP protein are low (i.e. in transgenic L lines and soon after transient expression of the reporter protein in *N*. *benthamiana* cells), the reduced accessibility of the endogenous DXR enzymes to external proteolytic degradation, and the co-localization of part of these enzymes with envelope membrane fractions (Figs 1, 2 and 3) further suggest that the vesicles could be formed by engulfing stromal fractions containing soluble (i.e. active) DXR proteins when the endogenous enzymes accumulate above a certain threshold. Eventually, the vesicles could be expelled from the chloroplast, perhaps to deliver their stromal content (including DXR-GFP) to degradation by a process likely independent of autophagy.

The role of autophagy in the removal of chloroplast proteins (e.g. via RCBs) and metabolites (e.g. via SSGLs) has been well established [48–50,54]. It was proposed that RCBs and SSGLs (i.e. vesicles containing stromal material) might be formed by sequestering chloroplast protrusions or stromules from the main body of the organelle by an isolation membrane [48,49]. Once released into the cytoplasm, these vesicles would be engulfed by autophagosomes and transported to the vacuole for degradation. In the case of DXR-GFP vesicles, our results suggest that they are formed inside the chloroplast by trapping stromal contents (phase #1) and then transported to the envelope (phase #2). Following an evagination process (phase #3), a vesicle likely surrounded by envelope membranes would be released from the chloroplast (phase #4). The destination of exported vesicles within the cell remains unknown. However, some of them were found in the vicinity of the vacuole (Fig 6), suggesting that they might eventually release their content into these organelles for degradation. Besides autophagy, other vesicle-mediated pathways have been described for the degradation of chloroplast stroma proteins, including those involving senescence-associated vacuoles (SAVs) [55] and CCVs [52]. In the case of CCVs, treatment with concanamycin A inhibited the release of CCVs from chloroplasts, similar to what we observed for DXR-GFP (Fig 7). However, further experiments would be necessary to ascertain the specific nature of the DXR-GFP vesicles.

Our work confirms that chloroplast protein homeostasis is a complex phenomenon achieved by multiple mechanisms. The functioning of chloroplasts is intimately integrated into the metabolism of plant cells but these endosymbiotic organelles still remain semi-autonomous functional entities that are able to regulate their own biochemistry by relatively independent mechanisms. An important part of this regulation relies on the effective control of plastidial enzyme activities. In the chloroplast, complex networks of plastidial chaperones ensure proper folding, assembly, and suborganellar targeting of imported proteins. Chaperones and proteases are also essential components of the protein quality control system that allows the stabilization, refolding, or degradation of mature proteins that lose their native conformation and activity after metabolic perturbations or environmental challenges [4–6]. We previously found that inactive (e.g. misfolded) forms of DXS are recognized by the Arabidopsis J-protein J20 and hence delivered to Hsp70 and Hsp100 chaperones for either proper folding or degradation by the Clp protease [30,31]. Here we show that an excess of DXR activity might trigger a completely different degradation pathway that involves the packaging of the enzyme in vesicles and its eventual removal, likely by an autophagy-independent process. Interestingly, gradual depletion of a catalytic subunit of the stromal Clp protease in the alga *Chlamydomonas reinhardtii* induces the formation of vesicles and a cytoplasmic vacuolization reminiscent of autophagy [56], suggesting a connection between intra- and extraplastidial mechanisms for stromal protein degradation. Together, our results suggest that distinct but likely coordinated mechanisms act to control DXS and DXR homeostasis in order to tightly regulate the metabolic flux of the MEP pathway. We propose that the localization of these enzymes in non-stromal fractions does not respond to a specific physiological role beyond the control of enzyme activity levels via aggregation/disaggregation and protein-specific degradation mechanisms. Understanding the molecular nature of these mechanisms during the normal plant life cycle will contribute to make more informed decisions in future biotechnological approaches aimed to manipulate the levels of plastidial isoprenoids of interest in crop plants.
