# Correction: Differential Subplastidial Localization and Turnover of Enzymes Involved in Isoprenoid Biosynthesis in Chloroplasts

**DOI:** 10.1371/journal.pone.0274982

**Published:** 2022-09-16

**Authors:** Catalina Perello, Ernesto Llamas, Vincent Burlat, Miriam Ortiz-Alcaide, Michael A. Phillips, Pablo Pulido, Manuel Rodriguez-Concepcion

There are errors in the Funding statement. The correct Funding statement is as follows: This work was funded by grants from Generalitat de Catalunya (2014SGR-1434), FEDER and Spanish Ministerio de Economia y Competitividad (BIO2014-59092-P and BIO2015-71703-REDT), and European Commission FP7 (TiMet, contract 245143) to MRC. CP and MOA received predoctoral fellowships from Spanish CSIC (JAE-PRE program) and MINECO (FPI program), respectively. EL is supported by a Mexican CONACYT predoctoral fellowship. The funders had no role in study design, data collection.
